# A Rare Case of Miller Fisher Syndrome Following Legionella Pneumonia: Expanding the Spectrum of Post-infectious Neurological Complications

**DOI:** 10.7759/cureus.91660

**Published:** 2025-09-05

**Authors:** Neil Garg, Jackson Ferris, Cecilia Canale, Sarkis Kouyoumjian

**Affiliations:** 1 Department of Neurology, Wayne State University School of Medicine, Detroit, USA; 2 Department of Psychiatry and Behavioral Neurosciences, Wayne State University School of Medicine, Detroit, USA; 3 Department of Emergency Medicine, Wayne State University School of Medicine, Detroit, USA

**Keywords:** demyelinating disease, geriatric patient, guillain-barré syndrome, legionella pneumophila, miller fisher syndrome, neurological autoimmune disorders

## Abstract

Miller Fisher syndrome (MFS) is a rare variant of Guillain-Barré syndrome (GBS), typically presenting with ophthalmoplegia, ataxia, and areflexia, most often after viral or bacterial infections. Legionella pneumophila has been reported as a trigger for GBS, but its association with MFS has not been clearly described. We present a 70-year-old man with a history of ankylosing spondylitis who was hospitalized for Legionella pneumonia and subsequently developed progressive weakness, paresthesias, dysarthria, ptosis, facial droop, and ophthalmoplegia over 10 days. Spinal imaging showed chronic cervical cord compression, but lumbar puncture revealed albuminocytologic dissociation consistent with GBS. Nerve conduction studies and electromyography were not performed; diagnosis was made on the basis of clinical features and CSF findings. Following initiation of intravenous immunoglobulin, his symptoms began improving within one week: dysarthria, ptosis, and ophthalmoplegia resolved, paresthesias disappeared, and limb strength improved on neurological examination. He was transferred to an inpatient rehabilitation facility, where mobility and strength further improved, allowing him to ambulate with a walker. By day 56, he returned to his neurological baseline with normal cranial nerve function, reflexes, and gait assisted by his walker. This case underscores a potential novel association between Legionella and MFS and expands the spectrum of post-infectious etiologies of demyelinating neuropathies.

## Introduction

We present a case of Miller Fisher syndrome (MFS), a rare autoimmune polyneuropathy and variant of Guillain-Barré syndrome (GBS) that was first described in 1956 and accounts for approximately 5% of GBS cases worldwide [1,2]. What makes this case particularly noteworthy is not only the uncommon neurological syndrome but also the unusual microbial trigger that led to its development. Like GBS, MFS arises from an aberrant immune response in which molecular mimicry following infection induces inflammatory demyelination [2,3]. The distinction of MFS lies in its clinical presentation; rather than the classic ascending paralysis seen in GBS, MFS is typically characterized by the triad of ataxia, areflexia, and ophthalmoplegia [1,4]. Symptoms usually begin within days to three weeks after infection, and diagnosis and monitoring often rely on objective measures such as neurological examination, cerebrospinal fluid (CSF) analysis, nerve conduction studies or electromyography, anti-GQ1b antibody testing, and validated clinical disability scores [2,4,5].

Most cases of GBS and MFS alike emerge as post-infectious neuropathies following an upper respiratory tract or gastrointestinal tract infection from well-established pathogens, including Campylobacter jejuni, cytomegalovirus, Epstein-Barr virus, and influenza [2,4]. The case below outlines the progressive course of MFS in a 70-year-old patient who presented with lower extremity weakness 10 days after an admission for Legionella pneumophila pneumonia, a gram-negative bacillus with only a handful of reported associations with GBS and its many variants.

## Case presentation

A 70-year-old Caucasian male with a history of ankylosing spondylitis, type 2 diabetes mellitus, hypertension, and prior cervical spinal fusion (C4-C6) presented to the hospital as an outside transfer on day 0. He presented with bilateral lower extremity weakness four days prior (day four). Significant home medications included adalimumab 40 mg biweekly, prednisone 10 mg daily, and fluticasone-salmeterol twice daily. He had recently been hospitalized for sepsis secondary to Legionella pneumophila pneumonia and treated with a seven-day course of azithromycin from days -14 to -6. From days -4 to 0, the patient developed bilateral lower extremity weakness and worsening back pain. He had previously ambulated with a walker but was now wheelchair-bound. His back pain was diffuse, constant, and worse with lying down. He also reported paresthesia, pain, and numbness in his fingers bilaterally. He denied bowel or bladder dysfunction or lower extremity paresthesias.

**Figure 1:**
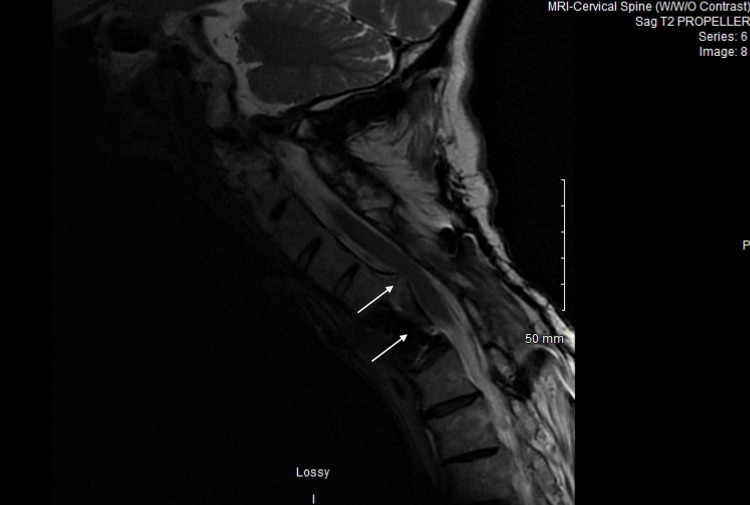
Sagittal T2-weighted MRI of the cervical spine demonstrating severe anterior cervical cord compression at the C4–C6 levels.

**Figure 2:**
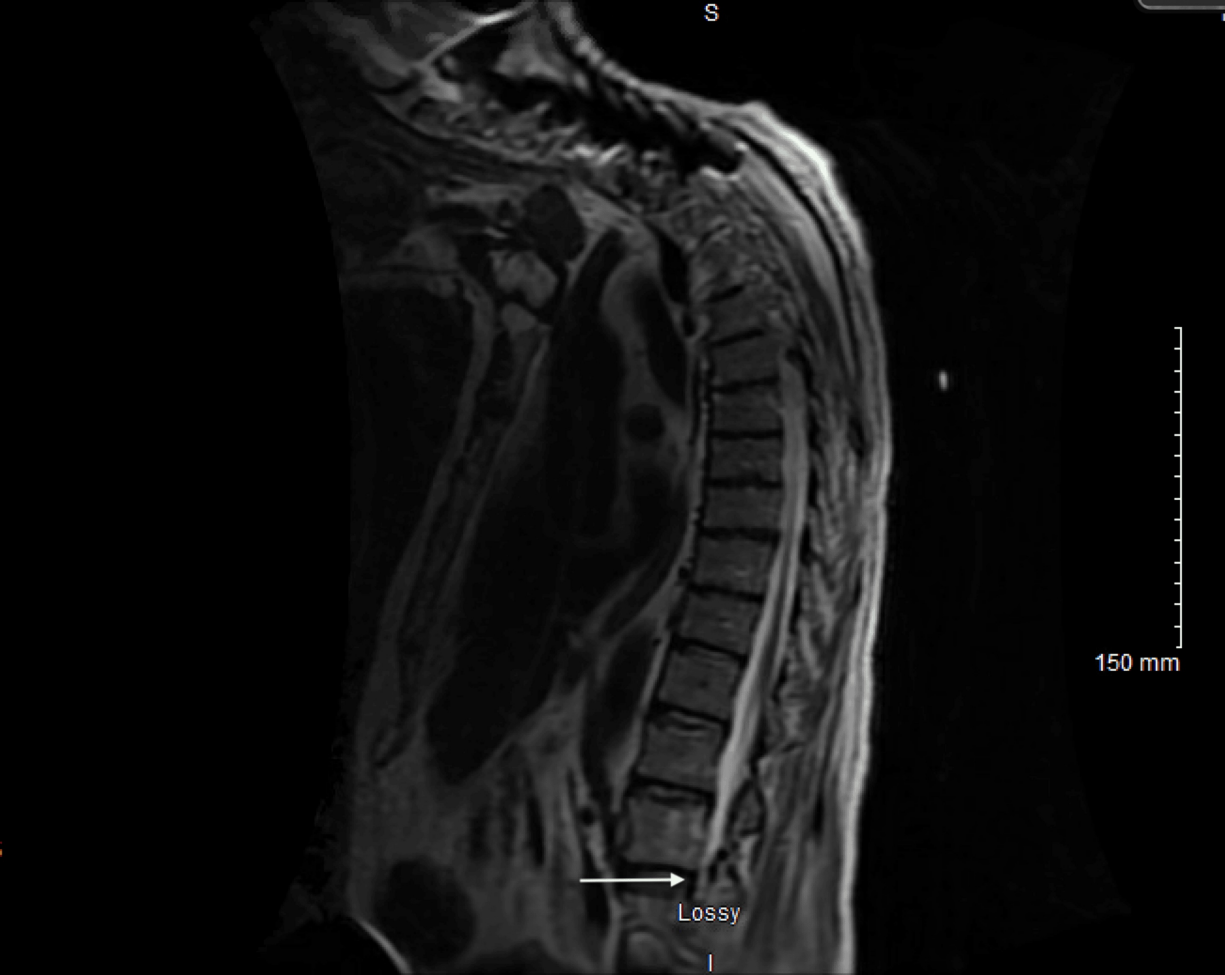
Sagittal T2-weighted MRI of the thoracic and lumbar spine with and without contrast, demonstrating mild-to-moderate foraminal narrowing from L3 to S1, consistent with chronic degenerative and inflammatory changes.

On arrival, he was hypertensive (176/122 mmHg), tachycardic (105 bpm), and afebrile (36.3°C). Due to concerns for spinal cord pathology, cervical and thoracolumbar spine MRI were performed. These images showed severe anterior cord compression at the lower cervical level (Figure 1), a bulging disc affecting the L5 nerve root, and mild-to-moderate foraminal narrowing from L3 to S1 consistent with chronic degenerative and inflammatory changes (Figure 2). At this time, spinal stenosis and cord compression were the leading differential diagnoses. Neurosurgery was consulted, and the patient was started on dexamethasone.

The following day (day one), the patient’s clinical status remained unchanged. Neurosurgery reviewed the imaging and determined that no surgical intervention was warranted, recommending continued steroid therapy. On day two, the patient began to develop dysarthria, prompting a stat non-contrast head CT to evaluate for stroke. Imaging revealed a prominent basilar tip (~6 mm in diameter), which was concerning for a possible aneurysm. Given the wide range of symptoms that a basilar tip aneurysm can present with, this was considered a potential explanation for the patient’s symptoms. However, a follow-up CT angiography of the head and neck definitively ruled out an aneurysm.

The patient’s clinical presentation worsened over days three to four. He developed left-sided ptosis, facial droop, ophthalmoplegia, burning sensations in the legs, worsening dysarthria, and bilateral upper extremity weakness. At that time, he showed no improvement on steroids. With stroke, aneurysm, and structural causes being ruled out, the clinical picture was now suggestive of an autoimmune demyelinating polyneuropathy. Thus, an autoimmune panel was sent, and a lumbar puncture was performed. The autoimmune panel showed an isolated increase in ANA, consistent with his ankylosing spondylitis. The lumbar puncture was significant for albuminocytologic dissociation (CSF protein = 171 mg/dL), consistent with GBS. The progressive clinical presentation following a respiratory infection, along with the CSF studies, confirmed the diagnosis of MFS. The neurological exam prior to treatment for MFS showed left-sided ptosis, facial droop, ophthalmoplegia, dysarthria, diffuse hyporeflexia, and varying degrees of strength loss: 5/5 in shoulder abduction/adduction; 4/5 in biceps flexion, triceps extension, plantarflexion, and dorsiflexion; 3/5 in finger extension/flexion, interossei activation, hip flexion/extension, and knee flexion/extension (Table 1). Plantar responses were flexor bilaterally.

**Table 1:** Serial motor strength examination from initial presentation (day 0) through treatment response (day 16) and follow-up (day 56).

Movement	Day 0		Day 16		Day 56	
	Left	Right	Left	Right	Left	Right
Shoulder abduction	5/5	5/5	5/5	5/5	5/5	5/5
Shoulder adduction	5/5	5/5	5/5	5/5	5/5	5/5
Biceps flexion	4/5	4/5	5/5	5/5	5/5	5/5
Triceps extension	4/5	4/5	5/5	5/5	5/5	5/5
Finger extension	3/5	3/5	4/5	4/5	5/5	5/5
Finger flexion	3/5	3/5	4/5	4/5	5/5	5/5
Interossei adduction/abduction	3/5	3/5	4/5	4/5	5/5	5/5
Hip flexion	3/5	3/5	4/5	4/5	4/5	4/5
Knee flexion	3/5	3/5	4/5	4/5	4/5	4/5
Knee extension	3/5	3/5	4/5	4/5	4/5	4/5
Plantarflexion	4/5	4/5	4/5	4/5	4/5	4/5
Dorsiflexion	4/5	4/5	4/5	4/5	4/5	4/5

On day five, the patient was started on intravenous immunoglobulin (IVIG) at 0.4 g/kg/day for five days. From days six to 16, serial neurological examinations demonstrated gradual improvement. Dysarthria resolved, ptosis and ophthalmoplegia resolved, and distal paresthesias were no longer present. Motor strength also improved, with shoulder and elbow movements returning to 5/5 bilaterally, finger movements improving from 3/5 to 4/5, and hip and knee strength increasing from 3/5 to 4/5 (Table 1). He progressed from wheelchair dependence to ambulating short distances with a walker. The patient was transferred to an inpatient rehabilitation facility, where he regained independence with transfers and advanced to longer distance ambulation. By day 56, neurological examination showed return to his pre-hospitalization baseline: cranial nerve function was intact, deep tendon reflexes were present, finger strength had improved to 5/5 bilaterally, and lower extremity strength remained 4/5 bilaterally, consistent with his chronic baseline due to prior cervical spine disease. He ambulated safely with the use of his walker (Table 1).

## Discussion

GBS classically affects Schwann cells, destroying the myelin sheath surrounding axons of the peripheral nervous system [3,5]. Its etiology is typically classified as either an acute inflammatory demyelinating polyneuropathy (AIDP) or an acute motor axonal neuropathy (AMAN). AIDP is characterized by T-cell-mediated Schwann cell demyelination, whereas AMAN involves axonal injury due to IgG and complement activation, with minimal T-cell mobilization [5]. GBS most commonly presents with an ascending pattern of symmetrical weakness and decreased deep tendon reflexes starting in the lower extremities [1,3,6]. Facial paralysis, respiratory failure, and autonomic dysregulation (blood pressure and heart rate fluctuations) can occur and must be monitored closely [6]. GBS is typically preceded by an upper respiratory infection, gastroenteritis, certain vaccinations, immune checkpoint inhibitors, ganglioside therapy, or surgery [5]. Infectious triggers have been heavily implicated in the development of GBS. The most common pathogens include Campylobacter jejuni, Haemophilus influenzae, Mycoplasma pneumoniae, cytomegalovirus, Epstein-Barr virus, hepatitis E virus, influenza A virus, Zika virus, dengue, chikungunya, and SARS-CoV-2 [5]. Legionella pneumophila, a gram-negative intracellular bacillus responsible for a type of atypical pneumonia, is a rare cause of GBS. Current literature reflects several case reports of Legionella-associated GBS, both in adults and pediatric patients [7-11]. Notably, Legionella infections occur most often in older adults, particularly those in communal or healthcare-associated settings, making recognition of such complications in this population clinically important [12].

MFS is a rare GBS variant that typically presents with bilateral ophthalmoparesis, severe ataxia, decreased tendon reflexes, facial nerve palsy, sensory deficits, and hyposthenia [1,3]. The pathogenesis of both GBS and MFS involves molecular mimicry, wherein an immune response to infection results in the formation of cross-reactive antibodies that target ganglioside carbohydrate moieties on peripheral nerve cells [5,6]. IgG antibodies against GM1a, GM1b, GD1a, and GalNAc-GD1a gangliosides have been associated with classical GBS, whereas anti-GQ1b antibodies, which are more specific to MFS, are the most commonly tested [6]. Current international guidelines emphasize that the diagnosis of GBS and its variants is primarily clinical, supported by cerebrospinal fluid analysis and electrodiagnostic studies, with antibody testing considered useful mainly in variants such as MFS [13].

In this case, the patient developed proximal lower extremity weakness that progressed distally over 10 days, accompanied by pain and paresthesias in the distal fingers, areflexia in all extremities, severe ataxia resulting in inability to ambulate, progressive dysarthria, and left-sided ptosis with ophthalmoparesis. These symptoms emerged 10 to 21 days after a Legionella-induced atypical pneumonia. These findings are consistent with MFS. Although Legionella-associated GBS has been described in prior reports, MFS as a manifestation remains distinctly uncommon. This case adds to the limited literature suggesting that Legionella can trigger not only classical GBS but also rare variants such as MFS.

One limitation of this case is the absence of confirmatory testing for anti-GQ1b antibodies, which are more specific to MFS. This testing would have required a send-out to an out-of-state laboratory and was not deemed necessary by current guidelines, particularly given the associated costs and the fact that the results would not alter the patient’s management [13]. Similarly, nerve conduction studies, electromyography, and validated clinical disability scoring were not performed, which modestly limits diagnostic certainty. International guidelines from the European Academy of Neurology and Peripheral Nerve Society emphasize that GBS and its variants are diagnosed clinically, supported by cerebrospinal fluid analysis and electrodiagnostic studies, with antibody testing considered supportive in variants such as MFS [13]. These guidelines also recommend validated scores such as the modified Erasmus GBS Outcome Score (mEGOS) and the modified Erasmus GBS Respiratory Insufficiency Score (mEGRIS) to assist in prognostication and monitoring, although these were not applied in this case [13].

The patient’s severe underlying spinal degenerative disease likely contributed to baseline sensory and motor deficits, complicating the assessment of early weakness. His history included mild-to-moderate foraminal narrowing from L3 to S1, consistent with chronic degenerative and inflammatory changes, severe anterior cervical cord compression at C4-C6, prior cervical spinal fusion at C4-C6, and ankylosing spondylitis. These conditions could account for chronic radicular pain, lower extremity weakness, gait instability, and sensory changes. However, the abrupt progression of weakness over 10 days, the presence of generalized areflexia, and the development of ophthalmoparesis and ataxia are not explained by chronic spinal pathology. The patient had been admitted to an outside hospital for sepsis secondary to Legionella pneumonia prior. Detailed records from that hospitalization, including ICU status, oxygen requirement, and chest imaging, were not available, which limits our ability to definitively exclude critical illness polyneuropathy. Additionally, given the patient’s history of adalimumab use, tumor necrosis factor inhibitor-associated neuropathy was considered. While these alternative etiologies remain plausible, the clinical picture, particularly the cranial nerve involvement and classic triad, most strongly supports MFS as a rare post-infectious manifestation of Legionella infection.

## Conclusions

This case describes a rare presentation of MFS following a Legionella pneumophila infection. While Legionella-associated GBS has been documented in prior reports, involvement of the MFS variant remains distinctly uncommon. This may be clinically relevant given that Legionella more commonly affects older adults, particularly those in communal or healthcare-associated settings. MFS can present with ataxia, diplopia, and facial weakness, symptoms that can be mistaken for stroke, myasthenia gravis, or age-related imbalance. This case broadens the differential diagnosis for post-infectious neurologic syndromes in elderly patients and highlights the importance of recognizing progressive neurological symptoms in the context of recent Legionella infection. Early identification is essential for accurate diagnosis, timely treatment, and optimal recovery to baseline neurological function.
